# MicroRNA-messenger RNA interactions involving JAK-STAT signaling genes in colorectal cancer

**DOI:** 10.18632/genesandcancer.177

**Published:** 2018-05

**Authors:** Lila E. Mullany, Jennifer S. Herrick, Lori C. Sakoda, Wade Samowitz, John R. Stevens, Roger K. Wolff, Martha L. Slattery

**Affiliations:** ^1^ Department of Medicine, University of Utah, 383 Colorow, Salt Lake City, Utah; ^2^ Division of Research, Kaiser Permanente Northern California, Oakland, California; ^3^ Department of Pathology, University of Utah, Salt Lake City, Utah; ^4^ Department of Mathematics and Statistics, Utah State University, Logan, Utah

**Keywords:** colorectal, cancer, JAK-STAT, miRNA, receptor

## Abstract

JAK-STAT signaling influences many downstream processes that, unchecked, contribute to carcinogenesis and metastasis. MicroRNAs (miRNAs) are hypothesized as a mechanism to prevent uncontrolled growth from continuous JAK-STAT activation. We investigated differential expression between paired carcinoma and normal colorectal mucosa of messenger RNAs (mRNAs) and miRNAs using RNA-Seq and Agilent Human miRNA Microarray V19.0 data, respectively, using a negative binomial mixed effects model to test 122 JAK-STAT-signaling genes in 217 colorectal cancer (CRC) cases. Overall, 42 mRNAs were differentially expressed with a fold change of >1.50 or <0.67, remaining significant with a false discovery rate of < 0.05; four were dysregulated in microsatellite stable (MSS) tumors, eight were for microsatellite unstable (MSI)-specific tumors. Of these 54 mRNAs, 17 were associated with differential expression of 46 miRNAs, comprising 116 interactions: 16 were significant overall, one for MSS tumors only. Twenty of the 29 interactions with negative beta coefficients involved miRNA seed sequence matches with mRNAs, supporting miRNA-mediated mRNA repression; 17 of these mRNAs encode for receptor molecules. Receptor molecule degradation is an established JAK-STAT signaling control mechanism; our results suggest that miRNAs facilitate this process. Interactions involving positive beta coefficients may illustrate downstream effects of disrupted STAT activity, and subsequent miRNA upregulation.

## INTRODUCTION

Janus Kinases (JAKs) and signal transducers and activators of transcription (STATs) are the central proteins of the JAK-STAT signaling pathway, which, when activated, is known to stimulate cell proliferation, differentiation, cell migration and apoptosis [1]. These processes are dysregulated in many diseases, including colorectal cancer (CRC), and the JAK-STAT signaling pathway has been implicated in CRC development and progression [2, 3]. We have previously identified single nucleotide polymorphisms within genes in the JAK-STAT signaling pathway that are associated significantly with risk of developing colon and rectal cancers [2].

The cytoplasmic domains of type I and II cytokine receptors do not have catalytic abilities [4], and thus they rely on JAKs to activate the signal transduction pathway. In mammals, there are four main JAK proteins: JAK1, JAK2, JAK3 and Tyrosine Kinase 2 (TYK2) [5], and seven STAT proteins: STAT1, STAT2, STAT3, STAT4, STAT5A, STAT5B and STAT6 [6]. JAK proteins belong to the non-receptor family of tyrosine kinases [3]. They are cytoplasmic enzymes that are able to transfer a phosphate molecule from an adenosine triphosphate molecule to a tyrosine residue of a protein [7]. JAKs associate with intracellular domain of receptor molecules [8]. Ligands, including cytokines, hormones and growth factors, bind to the receptor; the receptor undergoes a conformational change, bringing the JAKs in closer proximity [7]. The JAKs then undergo auto-and/or trans-phosphorylation of tyrosine residues [5]. This activation allows JAKs to then phosphorylate tyrosine residues in the cytoplasmic region of the receptor, creating a docking site for STAT proteins [7], which are latent transcription factors residing in the cytoplasm [1]. STATs, in an inactive dimer conformation, then attach to the phosphotyrosine residues on the receptors, becoming phosphorylated in turn by the activated JAKs [7]. The activated STAT dimer then translocates to the nucleus, where it increases transcriptional activity [9].

As previously stated, JAK-STAT activation stimulates cell proliferation as well as other processes that contribute to metastasis, and unchecked stimulation of this pathway may lead to uncontrolled cell growth. As such, JAK-STAT signaling is meant to be transient, and to ensure this there are various feedback mechanisms in place to halt the signaling process at various steps, including degradation of receptors, dephosphorylation of JAKs, introduction of suppressors of cytokines (SOCs) and inactivation of STAT dimers [6]. MicroRNAs (miRNAs), small, non-coding regulatory molecules, are able to alter mRNA expression by post-transcriptionally binding mRNA molecules, and either causing transcript degradation or inhibition of translation, and as such they have been implicated as a feedback mechanism of regulation of JAK-STAT signaling genes [6, 10, 11]. We have previously identified miRNAs associated with CRC stage at diagnosis and survival [12, 13], and miRNAs, including miR-21, miR-29a, miR-29b-1 and miR-155, have been shown by others to be induced by the JAK-STAT pathway [6]. In turn, the JAK-STAT pathway can be activated in various cell lines and model organisms by miRNAs, such as miR-19a [14] and miR-9 [15], through repression of mRNAs such as SOCs.

In this study, we identified JAK-STAT signaling genes that were differentially expressed between normal colorectal mucosa and colorectal carcinoma tissue, and subsequently tested associations between significantly differentially expressed mRNAs and miRNAs. We hypothesize that miRNAs are able to influence CRC through their regulation of genes involved in the JAK-STAT signaling pathway.

## RESULTS

The mean age of the study population was 64.8 years (Table 1). The majority of the samples were obtained from individuals diagnosed with colon cancer (77.9%); 22.1% of samples came from individuals diagnosed with rectal cancer. Slightly more than half (54.4%) of the population was male. The majority of participants (74.2%) were non-Hispanic white individuals, with the remainder of Hispanic (6.5%), non-Hispanic black (3.7%), or unknown (15.7%) race/ethnicity; 13.4% were MSI.

**Table 1:** Description of Study Population

		*N*	%
**Site**			
	Colon	169	77.9
	Rectal	48	22.1
**Sex**			
	Male	118	54.4
	Female	99	45.6
**Age**			
	Mean (SD)	64.8	10.1
**Race**			
	non-Hispanic White	161	74.2
	Hispanic	14	6.5
	non-Hispanic Black	8	3.7
	Unknown	34	15.7
**AJCC Stage**			
	1	58	27.1
	2	61	28.5
	3	72	33.6
	4	23	10.8
**Tumor Phenotype**			
	*TP53* mutated	103	47.5
	*KRAS* mutated	69	31.8
	*BRAF* mutated	21	10.1
	CIMP High	45	20.7
	MSI	29	13.4

In overall analyses, 93 JAK-STAT signaling genes were differentially expressed between carcinoma tissue and normal mucosa, of which 86 remained significantly associated after adjustment for multiple comparisons (Table 2). Forty-two of these 86 mRNAs had a FC >1.50 or <0.67. Considering only mRNAs that were differentially expressed with FC >1.50 or <0.67 and remained significant after adjustment for multiple comparisons, four mRNAs (*CNTF*, *PIAS2*, *SOCS6* and *IFNE*) were uniquely dysregulated in MSS tumors (Supplementary Table 2), and eight mRNAs (*IL21R*, *JAK3*, *CISH*, *GFAP*, *IL2RG*, *IL12A*, *LEP* and *HRAS*) had dysregulated expression for MSI-specific tumors (Supplementary Table 3). These 54 mRNAs were then subsequently tested for associations with differential miRNA expression for overall CRC, or in MSS or MSI tumors.

**Table 2:** Differential (carcinoma minus normal mucosa) mRNA expression of JAK-STAT signaling genes in colorectal cancer cases

	Mean Expression			P-values	
Gene Name	Carcinoma	Normal Mucosa	Fold Change	Raw	Adjusted
*AKT1*	153.83	152.34	1.01	0.708	0.738
*AKT2*	156.81	135.87	1.15	<0.001	<0.001
*AKT3*	41.45	52.56	0.79	<0.001	<0.001
***AOX1***	3.69	8.86	0.42	<0.001	<0.001
***BCL2***	24.42	62.11	0.39	<0.001	<0.001
***BCL2L1***	144.28	64.05	2.25	<0.001	<0.001
***CCND1***	317.79	122.64	2.59	<0.001	<0.001
***CCND2***	773.45	483.06	1.60	<0.001	<0.001
*CCND3*	33.57	40.72	0.82	<0.001	<0.001
*CDKN1A*	84.50	98.10	0.86	0.004	0.006
*CISH*	30.23	36.72	0.82	<0.001	<0.001
*CNTF*	3.26	4.86	0.67	<0.001	<0.001
***CNTFR***	0.73	4.85	0.15	<0.001	<0.001
*CREBBP*	264.23	273.44	0.97	0.061	0.079
*CSF2RA*	3.29	3.37	0.98	0.880	0.887
***CSF2RB***	27.82	73.57	0.38	<0.001	<0.001
***CSF3***	1.03	1.66	0.62	0.009	0.013
*CSF3R*	25.53	24.64	1.04	0.619	0.674
*CTF1*	1.33	1.11	1.20	0.254	0.302
*EP300*	293.90	339.29	0.87	<0.001	<0.001
***EPOR***	6.81	10.87	0.63	<0.001	<0.001
***FHL1***	21.10	96.69	0.22	<0.001	<0.001
*GFAP*	0.46	0.55	0.84	0.308	0.351
***GHR***	6.68	20.21	0.33	<0.001	<0.001
*GRB2*	114.66	112.16	1.02	0.407	0.455
*HRAS*	18.52	12.37	1.50	<0.001	<0.001
*IFNAR1*	100.68	99.38	1.01	0.675	0.716
*IFNAR2*	49.46	53.08	0.93	0.044	0.060
*IFNE*	0.65	0.36	1.83	0.038	0.052
***IFNG***	0.78	2.48	0.32	<0.001	<0.001
*IFNGR1*	49.74	44.34	1.12	0.004	0.006
*IFNGR2*	71.20	57.91	1.23	<0.001	<0.001
***IFNK***	5.85	11.76	0.50	<0.001	<0.001
*IL10*	0.82	1.04	0.79	0.137	0.173
***IL10RA***	47.02	118.37	0.40	<0.001	<0.001
*IL10RB*	38.15	54.43	0.70	<0.001	<0.001
***IL11***	4.99	1.58	3.16	<0.001	<0.001
***IL11RA***	11.35	21.84	0.52	<0.001	<0.001
*IL12A*	0.66	0.94	0.71	0.049	0.065
*IL12B*	0.30	0.49	0.62	0.068	0.087
*IL12RB1*	5.54	7.02	0.79	0.019	0.028
*IL12RB2*	6.45	6.76	0.95	0.575	0.632
***IL13***	0.21	0.58	0.36	<0.001	<0.001
*IL13RA1*	185.64	145.89	1.27	<0.001	<0.001
*IL13RA2*	0.30	0.52	0.58	0.037	0.051
***IL15***	17.83	28.58	0.62	<0.001	<0.001
*IL15RA*	18.50	15.76	1.17	0.008	0.012
***IL17D***	12.16	4.99	2.44	<0.001	<0.001
*IL19*	0.20	0.34	0.60	0.047	0.063
***IL20RA***	30.28	12.37	2.45	<0.001	<0.001
*IL20RB*	4.98	6.23	0.80	0.004	0.006
*IL21R*	8.58	10.26	0.84	0.048	0.064
*IL22RA1*	40.43	33.11	1.22	0.001	0.002
***IL22RA2***	0.32	0.66	0.48	0.003	0.004
*IL23A*	2.06	1.59	1.30	0.036	0.051
***IL23R***	2.97	5.83	0.51	<0.001	<0.001
***IL24***	3.15	8.15	0.39	<0.001	<0.001
*IL27RA*	10.05	10.24	0.98	0.817	0.838
*IL28RA*	26.18	31.15	0.84	0.002	0.004
*IL2RA*	7.04	6.38	1.10	0.280	0.325
*IL2RB*	17.40	20.32	0.86	0.034	0.048
*IL2RG*	44.20	50.55	0.87	0.027	0.039
***IL3RA***	2.21	3.59	0.62	0.003	0.005
*IL4R*	114.37	143.74	0.80	<0.001	<0.001
***IL5***	1.26	2.00	0.63	0.001	0.002
***IL5RA***	0.49	1.94	0.25	<0.001	<0.001
*IL6*	4.04	3.08	1.31	0.062	0.079
***IL6R***	25.93	101.24	0.26	<0.001	<0.001
***IL6ST***	166.16	281.27	0.59	<0.001	<0.001
*IL7*	15.39	15.71	0.98	0.741	0.766
***IL7R***	35.03	79.98	0.44	<0.001	<0.001
*IRF9*	61.65	61.12	1.01	0.839	0.853
*JAK1*	200.53	198.85	1.01	0.701	0.738
*JAK2*	46.91	57.44	0.82	<0.001	<0.001
*JAK3*	47.72	66.78	0.71	<0.001	<0.001
*LEP*	0.87	1.16	0.75	0.152	0.189
*LEPR*	11.97	14.69	0.81	0.004	0.006
***LIF***	75.84	44.62	1.70	<0.001	<0.001
***LIFR***	13.28	59.86	0.22	<0.001	<0.001
*MCL1*	615.27	794.46	0.77	<0.001	<0.001
***MPL***	0.90	1.60	0.56	<0.001	<0.001
*MTOR*	172.49	189.52	0.91	0.001	0.002
***MYC***	181.11	49.00	3.70	<0.001	<0.001
***OSM***	6.68	2.31	2.90	<0.001	<0.001
*OSMR*	39.06	47.83	0.82	<0.001	<0.001
*PIAS1*	38.62	47.06	0.82	<0.001	<0.001
*PIAS2*	26.48	37.23	0.71	<0.001	<0.001
*PIAS3*	36.31	34.92	1.04	0.357	0.403
*PIAS4*	29.27	35.75	0.82	<0.001	<0.001
*PIK3CA*	67.11	55.23	1.22	<0.001	<0.001
*PIK3CB*	90.89	81.36	1.12	0.001	0.001
***PIK3CD***	27.33	46.48	0.59	<0.001	<0.001
*PIK3R1*	154.42	149.09	1.04	0.300	0.345
*PIK3R2*	72.83	54.55	1.34	<0.001	<0.001
*PIK3R3*	35.88	33.87	1.06	0.255	0.302
*PIM1*	38.42	42.83	0.90	0.030	0.044
*PRLR*	100.62	93.37	1.08	0.216	0.264
***PTPN11***	227.06	116.88	1.94	<0.001	<0.001
*PTPN2*	31.73	31.08	1.02	0.674	0.716
*PTPN6*	38.91	47.34	0.82	<0.001	<0.001
*RAF1*	131.53	147.29	0.89	<0.001	<0.001
*SOCS1*	4.19	3.98	1.05	0.552	0.612
***SOCS2***	7.99	13.77	0.58	<0.001	<0.001
*SOCS3*	67.43	81.65	0.83	0.001	0.002
*SOCS4*	63.55	54.54	1.17	<0.001	<0.001
*SOCS5*	37.44	32.53	1.15	<0.001	0.001
*SOCS6*	47.54	69.67	0.68	<0.001	<0.001
***SOCS7***	22.20	14.71	1.51	<0.001	<0.001
*SOS1*	159.37	164.61	0.97	0.224	0.270
*SOS2*	91.40	127.77	0.72	<0.001	<0.001
*STAM*	35.53	26.52	1.34	<0.001	<0.001
*STAM2*	60.45	73.55	0.82	<0.001	<0.001
***STAT1***	341.01	220.37	1.55	<0.001	<0.001
*STAT2*	159.86	162.27	0.99	0.642	0.693
*STAT3*	228.06	249.88	0.91	<0.001	<0.001
***STAT4***	7.35	14.04	0.52	<0.001	<0.001
*STAT5A*	37.96	38.02	1.00	0.974	0.974
*STAT5B*	96.43	93.35	1.03	0.278	0.325
*STAT6*	257.47	264.74	0.97	0.176	0.217
***THPO***	0.58	1.00	0.58	0.006	0.009
***TSLP***	0.64	0.97	0.66	0.033	0.047
*TYK2*	124.25	147.05	0.84	<0.001	<0.001

**Bolded** genes are those with a fold change >1.5 or <0.67 and an adjusted p-value <0.05.

Seventeen mRNAs were associated significantly after adjustment for multiple comparisons with at least one miRNA that had a fold change >1.50 or <0.67 (Table 3). Differential expression of sixteen mRNAs was associated with miRNA differential expression for overall CRC; differential expression of *CNTF* was associated significantly with differential expression of one miRNA, hsa-miR-518c-5p, in MSS tumors only. There were no significant miRNA-mRNA associations for MSI tumors only. These findings comprised 116 unique miRNA-mRNA associations and 46 unique miRNAs. Of these interactions, 87 had a positive beta coefficient, indicating that as differential of the mRNA increased, differential expression of the miRNA increased as well. Conversely, 29 interactions displayed a negative beta coefficient, indicating that as either miRNA or mRNA differential expression increased, mRNA or miRNA differential expression decreased. Of the 116 mRNA-miRNA associations identified, 69 (59%) had a seed match between the miRNA and mRNA, supporting a direct interaction between the miRNA and mRNA.

**Table 3:** MiRNA-mRNA associations for differential (carcinoma minus normal mucosa) expression in colorectal cancer cases

	Mean Expression				Mean Expression				P-values	
Gene	Carcinoma	Normal Mucosa	Fold Change	miRNA	Carcinoma	Normal Mucosa	Fold Change	Beta	Raw	Adjusted
*BCL2*	24.42	62.11	0.39	**hsa-miR-150-5p**	14.90	39.17	0.38	0.34	<.0001	0.020
				**hsa-miR-195-5p**	3.59	12.18	0.29	0.24	0.000	0.049
				**hsa-miR-203a**	12.52	3.70	3.38	−0.27	<.0001	0.020
				**hsa-miR-650**	4.51	16.60	0.27	0.38	<.0001	0.020
*BCL2L1*	144.28	64.05	2.25	hsa-miR-92a-3p	121.60	41.18	2.95	0.34	<.0001	0.041
*CCND1*	317.79	122.64	2.59	**hsa-miR-106b-5p**	15.90	5.19	3.06	0.25	0.001	0.035
				**hsa-miR-17-5p**	61.04	16.38	3.73	0.30	<.0001	0.024
				**hsa-miR-19b-3p**	29.80	10.42	2.86	0.28	0.000	0.024
				hsa-miR-203a	12.52	3.70	3.38	0.27	0.000	0.024
				**hsa-miR-20a-5p**	70.78	17.61	4.02	0.28	<.0001	0.024
				**hsa-miR-20b-5p**	17.65	3.30	5.35	0.29	<.0001	0.024
				hsa-miR-21-5p	463.11	167.37	2.77	0.25	0.001	0.035
				**hsa-miR-221-3p**	13.53	4.12	3.28	0.26	0.000	0.024
				**hsa-miR-27a-3p**	56.26	23.29	2.42	0.27	0.000	0.024
				**hsa-miR-29b-3p**	24.31	9.83	2.47	0.27	0.000	0.024
				**hsa-miR-93-5p**	41.72	15.20	2.74	0.26	0.000	0.024
*CNTF*^1^	3.24	4.87	0.67	**hsa-miR-518c-5p**	1.76	2.90	0.61	0.27	<.0001	0.041
*CSF2RB*	27.82	73.57	0.38	**hsa-miR-1203**	1.76	2.83	0.62	0.23	0.001	0.050
				**hsa-miR-124-3p**	0.90	2.40	0.38	0.25	0.001	0.037
				**hsa-miR-150-5p**	14.90	39.17	0.38	0.30	<.0001	0.010
				hsa-miR-2117	1.50	4.09	0.37	0.28	0.000	0.024
				hsa-miR-3124-5p	1.37	2.27	0.60	0.28	<.0001	0.010
				hsa-miR-4315	0.21	2.62	0.08	0.23	0.001	0.050
				hsa-miR-4469	1.11	2.41	0.46	0.22	0.001	0.050
				hsa-miR-525-5p	1.56	2.53	0.62	0.22	0.001	0.050
				**hsa-miR-650**	4.51	16.60	0.27	0.37	<.0001	0.010
				**hsa-miR-92a-3p**	121.60	41.18	2.95	−0.24	0.001	0.050
*FHL1*	21.10	96.69	0.22	**hsa-miR-133b**	1.71	6.94	0.25	0.39	<.0001	0.016
				hsa-miR-145-5p	132.97	223.14	0.60	0.43	<.0001	0.016
				hsa-miR-193b-3p	9.12	5.42	1.68	0.24	0.000	0.041
				**hsa-miR-195-5p**	3.59	12.18	0.29	0.26	<.0001	0.016
				hsa-miR-30a-5p	2.38	4.61	0.52	0.23	0.001	0.048
*IL10RA*	47.02	118.37	0.40	**hsa-miR-106b-5p**	15.90	5.19	3.06	−0.23	0.001	0.044
				**hsa-miR-150-5p**	14.90	39.17	0.38	0.46	<.0001	0.008
				**hsa-miR-17-5p**	61.04	16.38	3.73	−0.25	0.001	0.023
				**hsa-miR-195-5p**	3.59	12.18	0.29	0.25	0.000	0.013
				hsa-miR-203a	12.52	3.70	3.38	−0.25	0.000	0.016
				**hsa-miR-20a-5p**	70.78	17.61	4.02	−0.23	0.001	0.044
				**hsa-miR-20b-5p**	17.65	3.30	5.35	−0.26	0.000	0.016
				hsa-miR-221-3p	13.53	4.12	3.28	−0.24	0.000	0.013
				hsa-miR-3651	58.66	25.92	2.26	−0.22	0.001	0.044
				**hsa-miR-429**	13.33	8.29	1.61	−0.31	<.0001	0.008
				**hsa-miR-650**	4.51	16.60	0.27	0.46	<.0001	0.008
				**hsa-miR-93-5p**	41.72	15.20	2.74	−0.25	0.001	0.030
*IL24*	3.15	8.15	0.39	hsa-miR-150-5p	14.90	39.17	0.38	0.33	<.0001	0.041
*IL6R*	25.93	101.24	0.26	**hsa-miR-150-5p**	14.90	39.17	0.38	0.30	<.0001	0.014
				**hsa-miR-17-5p**	61.04	16.38	3.73	−0.24	0.001	0.036
				**hsa-miR-19b-3p**	29.80	10.42	2.86	−0.30	0.000	0.014
				hsa-miR-203a	12.52	3.70	3.38	−0.23	0.001	0.034
				**hsa-miR-20a-5p**	70.78	17.61	4.02	−0.24	0.000	0.016
				**hsa-miR-20b-5p**	17.65	3.30	5.35	−0.29	0.000	0.014
				**hsa-miR-21-5p**	463.11	167.37	2.77	−0.29	<.0001	0.014
				**hsa-miR-2117**	1.50	4.09	0.37	0.22	0.001	0.039
				**hsa-miR-221-3p**	13.53	4.12	3.28	−0.22	0.002	0.049
				**hsa-miR-23a-3p**	174.68	87.53	2.00	−0.25	0.001	0.034
				**hsa-miR-27a-3p**	56.26	23.29	2.42	−0.28	<.0001	0.014
				**hsa-miR-3651**	58.66	25.92	2.26	−0.25	0.000	0.016
				**hsa-miR-518c-5p**	1.76	2.90	0.61	0.22	0.001	0.048
				**hsa-miR-650**	4.51	16.60	0.27	0.29	0.000	0.014
				hsa-miR-92a-3p	121.60	41.18	2.95	−0.28	<.0001	0.014
*IL6ST*	166.16	281.27	0.59	**hsa-miR-150-5p**	14.90	39.17	0.38	0.33	<.0001	0.041
				**hsa-miR-195-5p**	3.59	12.18	0.29	0.28	0.000	0.041
				**hsa-miR-497-5p**	1.77	7.12	0.25	0.25	0.000	0.049
				hsa-miR-650	4.51	16.60	0.27	0.27	<.0001	0.041
*IL7R*	35.03	79.98	0.44	**hsa-miR-150-5p**	14.90	39.17	0.38	0.32	<.0001	0.027
				hsa-miR-203a	12.52	3.70	3.38	−0.31	<.0001	0.027
				**hsa-miR-650**	4.51	16.60	0.27	0.26	<.0001	0.027
*LIFR*	13.28	59.86	0.22	**hsa-miR-133b**	1.71	6.94	0.25	0.26	0.000	0.019
				hsa-miR-145-5p	132.97	223.14	0.60	0.29	<.0001	0.009
				**hsa-miR-150-5p**	14.90	39.17	0.38	0.36	<.0001	0.009
				**hsa-miR-193b-3p**	9.12	5.42	1.68	0.23	0.001	0.043
				hsa-miR-195-5p	3.59	12.18	0.29	0.29	<.0001	0.009
				**hsa-miR-203a**	12.52	3.70	3.38	−0.31	0.000	0.015
				**hsa-miR-30a-5p**	2.38	4.61	0.52	0.27	<.0001	0.009
				hsa-miR-497-5p	1.77	7.12	0.25	0.33	<.0001	0.009
				hsa-miR-650	4.51	16.60	0.27	0.31	0.000	0.015
				hsa-miR-99a-5p	6.30	3.70	1.71	0.24	0.001	0.025
*MYC*	181.11	49.00	3.70	hsa-miR-1246	629.21	412.81	1.52	0.27	0.000	0.016
				hsa-miR-17-5p	61.04	16.38	3.73	0.35	<.0001	0.014
				hsa-miR-19b-3p	29.80	10.42	2.86	0.27	0.000	0.016
				hsa-miR-20a-5p	70.78	17.61	4.02	0.33	<.0001	0.014
				hsa-miR-20b-5p	17.65	3.30	5.35	0.31	0.000	0.016
				hsa-miR-3651	58.66	25.92	2.26	0.28	0.000	0.019
				hsa-miR-375	20.50	54.53	0.38	−0.29	<.0001	0.014
				hsa-miR-501-3p	7.07	2.95	2.39	0.26	0.000	0.019
				hsa-miR-583	6.61	3.22	2.05	0.26	0.000	0.023
				hsa-miR-663a	374.83	234.91	1.60	0.28	0.000	0.019
				hsa-miR-663b	65.50	32.21	2.03	0.33	<.0001	0.014
				hsa-miR-92a-3p	121.60	41.18	2.95	0.32	<.0001	0.014
*OSM*	6.68	2.31	2.90	hsa-miR-424-3p	39.81	25.37	1.57	−0.32	<.0001	0.041
				hsa-miR-934	4.36	0.94	4.66	0.25	<.0001	0.041
*PIK3CD*	27.33	46.48	0.59	**hsa-miR-150-5p**	14.90	39.17	0.38	0.35	<.0001	0.027
				**hsa-miR-650**	4.51	16.60	0.27	0.31	<.0001	0.027
*PTPN11*	227.06	116.88	1.94	hsa-miR-106b-5p	15.90	5.19	3.06	0.30	<.0001	0.008
				**hsa-miR-1246**	629.21	412.81	1.52	0.22	0.001	0.039
				hsa-miR-150-5p	14.90	39.17	0.38	−0.24	0.001	0.025
				hsa-miR-17-5p	61.04	16.38	3.73	0.32	<.0001	0.008
				**hsa-miR-195-5p**	3.59	12.18	0.29	−0.24	0.001	0.025
				**hsa-miR-19b-3p**	29.80	10.42	2.86	0.29	0.000	0.017
				**hsa-miR-203a**	12.52	3.70	3.38	0.23	0.001	0.029
				hsa-miR-20a-5p	70.78	17.61	4.02	0.30	<.0001	0.008
				hsa-miR-20b-5p	17.65	3.30	5.35	0.34	<.0001	0.008
				**hsa-miR-21-3p**	22.68	9.89	2.29	0.26	0.001	0.029
				**hsa-miR-21-5p**	463.11	167.37	2.77	0.27	0.000	0.017
				**hsa-miR-221-3p**	13.53	4.12	3.28	0.29	<.0001	0.008
				**hsa-miR-25-3p**	30.05	12.78	2.35	0.28	<.0001	0.008
				hsa-miR-27a-3p	56.26	23.29	2.42	0.26	0.001	0.033
				hsa-miR-29b-3p	24.31	9.83	2.47	0.24	0.001	0.029
				**hsa-miR-32-3p**	4.74	2.81	1.68	0.22	0.001	0.031
				**hsa-miR-34a-5p**	25.15	12.32	2.04	0.23	0.001	0.035
				hsa-miR-3651	58.66	25.92	2.26	0.30	<.0001	0.008
				**hsa-miR-425-5p**	11.76	6.97	1.69	0.29	<.0001	0.008
				**hsa-miR-650**	4.51	16.60	0.27	−0.31	<.0001	0.008
				**hsa-miR-92a-3p**	121.60	41.18	2.95	0.27	0.000	0.017
				hsa-miR-93-5p	41.72	15.20	2.74	0.30	<.0001	0.008
*STAT1*	341.01	220.37	1.55	**hsa-miR-146b-5p**	4.46	2.67	1.67	0.31	<.0001	0.016

**Bolded** miRNAs have an identified seed match with the associated mRNA.

1Significant for MSS tumors only.

**Figure 1:**
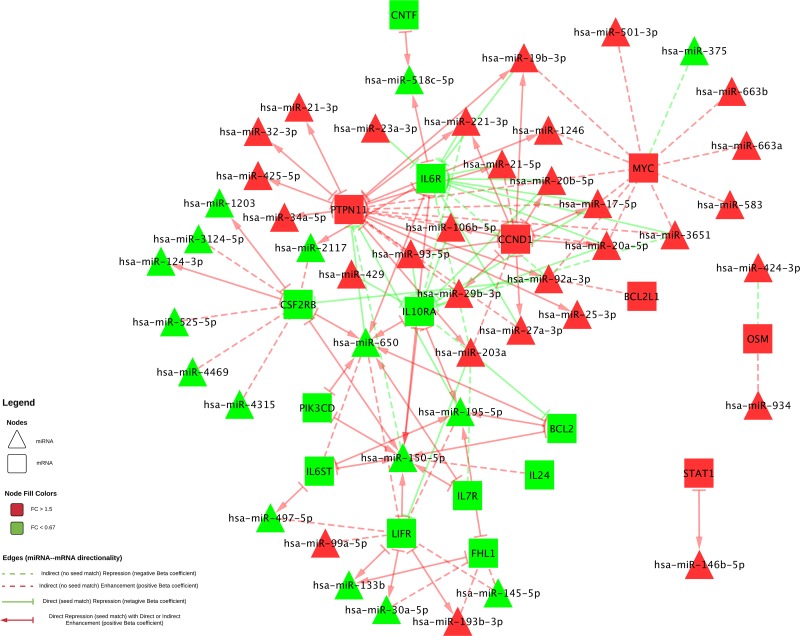
All significant miRNA-mRNA associations In Figure 1, dashed connecting lines signify interactions where a seed match was not identified, while solid lines with a stop (--|) indicate a seed match was found. The color of the connecting lines signifies the direction of the beta coefficient: a green line indicates a negative beta coefficient, and a red indicates a positive beta coefficient. An arrow (→) going from the mRNA to the miRNA is used in cases where a seed match was identified along with a positive beta coefficient. FC of the miRNA or mRNA is represented in the color of the molecule: green molecules have less expression in carcinoma tissue compared to normal mucosa and red have higher.

As shown in Figure 1, there are four types of miRNA-mRNA interactions, determined by the beta coefficient and whether a seed match between the miRNA and mRNA was identified. A negative beta coefficient combined with a seed match supports the classification of the miRNA-mRNA relationship as that of a canonical miRNA repression of the mRNA. Such interactions are displayed in Figure 1 with a solid green line and a stop (--|). Twenty total interactions (comprising six mRNAs and 16 miRNAs) displayed this type of relationship: *BCL2* with one miRNA (hsa-miR-203a), *CSF2RB* with one miRNA (hsa-miR-92a-3p), *IL10RA* with six miRNAs (hsa-miR-106b-5p, hsa-miR-17-5p, hsa-miR-20a-5p, hsa-miR-20b-5p, hsa-miR-429 and hsa-miR-93-5p), *IL6R* with nine miRNAs (hsa-miR-17-5p, hsa-miR-19b-3p, hsa-miR-20a/b-5p, hsa-miR-21-5p, hsa-miR-221-3p, hsa-miR-23a-3p, hsa-miR-27a-3p and hsa-miR-3651), *LIFR* with one miRNA (hsa-miR-203a) and *PTPN11* with two miRNAs (hsa-miR-195-5p and hsa-miR-650).

The smallest group of interactions comprised those that displayed a negative beta coefficient but no seed match identified between the miRNA and mRNA. These are shown in Figure 1 with a green dashed line. There were nine such interactions, involving six unique mRNAs (*OSM*, *IL7R*, *MYC*, *IL6R*, *IL10RA* and *PTPN11*) and seven unique miRNAs (hsa-miR-150-5p, hsa-miR-203a, hsa-miR-221-3p, hsa-miR-3651, hsa-miR-375, hsa-miR-424-3p and hsa-miR-92a-3p).

Thirty-eight interactions, comprising 10 mRNAs and 31 miRNAs, had a positive beta coefficient and no identified seed match; twenty of these miRNAs were involved with other mRNAs in interactions in which we identified a negative beta coefficient, a seed match, or both. These are shown in Figure 1 with a red dashed line.

Forty-nine interactions, between 13 mRNAs and 30 miRNAs, had identified seed matches but displayed a positive beta coefficient. These interactions are shown with a solid red line that has an arrow on one end, to indicate the positive beta coefficient, and a stop on the other, indicating the identified seed match (--|).

## DISCUSSION

Our paired mRNA and miRNA data are an asset to our investigation. Analyzing differential tissue expression enables identification of important expression changes in carcinoma tissue at the population level by using paired normal mucosa expression as a control for variations in individual samples that might occur during collection or processing. Our large miRNA platform facilitates a discovery-based approach, while maintaining high repeatability and reliability [16]. Although our study includes a relatively small number of CRC cases (*N* = 217), it is much larger than most existing studies containing both mRNA and miRNA data.

We analyzed expression levels of 122 genes involved in the JAK-STAT signaling pathway. Limiting our data to only those genes that were differentially expressed with a FC >1.50 or <0.67 and remained significantly associated after adjustment for multiple comparisons, 54 mRNAs were significantly differentially expressed: 42 in overall CRC, four in MSS-specific tumors and eight in MSI-specific tumors. Sixteen of these genes were associated with differential expression of 45 miRNAs, yielding 115 unique interactions in overall CRC. One mRNA, *CNTF*, was associated with differential expression of one miRNA, hsa-miR-518c-5p, in MSS tumors only. A match between the seed region of the miRNA and the 3′ UTR of the mRNA was identified for 69 of these total miRNA-mRNA interactions, supporting a greater likelihood of a direct involvement between the miRNA and mRNA.

Twenty miRNA-mRNA interactions displayed a negative beta coefficient and had an identified seed match, representing a canonical relationship of miRNA-mediated repression of mRNA expression. Nine miRNA-mRNA interactions had negative beta coefficients and no seed match. It is possible that these miRNAs repress these mRNAs and a seed match meeting criteria other than what we used in this analysis exists, or it is possible these associations are representative of indirect effects. The third group, comprising 38 interactions, had a positive beta coefficient and no identified seed match, suggesting that these associations reflect indirect or downstream effects, possibly leading to miRNA activation. Finally, 49 interactions, between 13 mRNAs and 30 miRNAs, had identified seed matches but displayed a positive beta coefficient. As seed matches were identified using the mRNA 3′ UTR and the first six to eight nucleotides on the 5′ end of the miRNA, this match supports a repressive action on the mRNA by the miRNA, which should result in less mRNA expression with increased miRNA expression. As this is not the case in our dataset, and we see increased (or decreased) carcinoma mRNA expression as carcinoma miRNA expression increased (or decreased), there may be additional influences on either the miRNA or mRNA expression. It is also possible that, in cases such as with *STAT1* and hsa-miR-146b-5p, this type of miRNA-mRNA interaction represents a feedback loop. As STAT1 is a transcription activator, it is possible that the positive beta coefficient reflects enhancement hsa-miR-146b-5p transcription by STAT1. The seed match between this pair indicates that *STAT1* may be targeted by hsa-miR-146b-5p in turn. It may also be that the binding may affect translation but have no effect on mRNA stability, which could explain why we do not detect a negative beta coefficient.

As we only looked at mRNA expression, rather than protein expression, we are unable to determine if mRNA translation is being inhibited, only if mRNA transcript presence is lessened, either by less transcription or transcript degradation by miRNAs. In cases of a positive beta coefficient it is therefore possible that mRNA transcription could be increasing, but incomplete miRNA binding prevents these transcripts from being translated. It is also possible that the miRNAs are responsible for increased levels of mRNA transcript production. While the canonical mechanism of miRNA-mRNA is translational repression of the mRNA by the miRNA within the cytoplasm, it has been proposed more recently that they may also be able to enhance translation [17]. Additionally, the same machinery that regulates post-transcriptional mRNA degradation and inhibition may participate in transcriptional control, enabling miRNAs to upregulate transcription of mRNAs [18–21].

We compared our results to miRTarBase [22] to identify miRNA-mRNA associations that have been experimentally validated. Of our 116 unique interactions, 14 were also in miRTarBase. One such interaction, between *MYC* and hsa-miR-375, had a negative beta coefficient, but no identified seed match. Three interactions, between *MYC* and hsa-miR-92a-3p, hsa-miR-20a-5p and hsa-miR-17-5p, had a positive beta coefficient and no seed match. Two interactions, between *LIFR* and hsa-miR-203a and *IL6R* and hsa-miR-23a-3p, had a negative beta coefficient and a seed match. Mir-23a and miR-23b are downregulated in prostate cancer cells and have been shown to target *IL6R*, and as such have been proposed as therapeutic targets for prostate cancer [23]. We did not detect significant changes in expression with miR-23b and mir-23a-3p was upregulated in colorectal carcinoma tissue compared to normal colorectal mucosa, however the negative association between miR-23-a-3p and *IL6R* along with an identified seed match supports the findings by Aghaee-Bakhtiari et al. Eight interactions that had positive beta coefficients and an identified seed match have been experimentally verified: between *BCL2* and hsa-miR-195-5p, between *CCND1* and hsa-miR-106b-5p, hsa-miR-93-5p, hsa-miR-27a-3p, hsa-miR-20a-5p, hsa-miR-20b-5p and hsa-miR-17-5p, and between *LIFR* and hsa-miR-30a-5p. That only 14 of the associations have been experimentally verified is most likely due to bias in the existing literature, as interactions with either positive or negative beta coefficients, and with an identified seed match or not, have been experimentally verified. It is also possible that different tissues were used for these associations, or different criteria for a seed sequence would have identified a match in our dataset.

**Figure 2:**
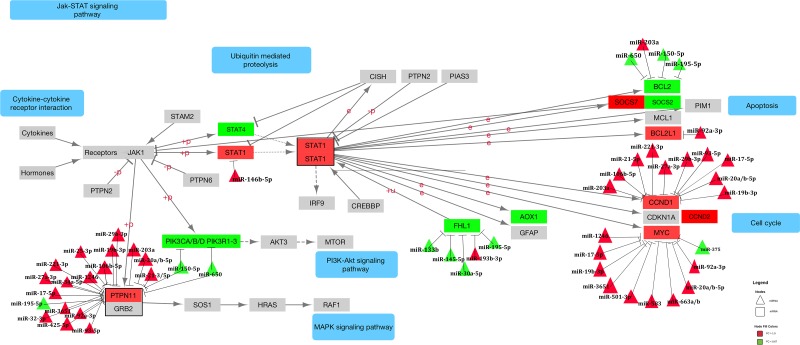
Dysregulated genes and associated miRNAs within the JAK-STAT signaling pathway Figure 2 displays the JAK-STAT signaling KEGG pathway as well as the miRNAs found to be associated significantly with the mRNA. All miRNA-mRNA associations are displayed with a stop (--|), regardless of whether or not a seed match was identified. FC of the miRNA or mRNA is represented in the color of the molecule: green molecules have less expression in carcinoma tissue compared to normal mucosa and red have higher.

Of the 42 mRNAs that were differentially expressed for overall CRC with a FC >1.50 or <0.67, 16 encoded for receptor proteins, 11 encoded for cytokines, two encoded for hormones, two encoded for STATs (*STAT1* and *STAT4*) and two for SOCs (*SOCS2* and *SOCS7*). The remaining nine genes encoded for downstream proteins: *AOX1*, *BCL2*, *BCL2L1*, *CCND1*/*2*, *FHL1*, *MYC*, *PIK3CD* and *PTPN11* (see Figure 2). Of the mRNAs associated significantly with differential miRNA expression, *PTPN11* was associated with the most miRNAs, with 22 interactions. *IL6R* was associated with 15 miRNAs, *MYC* and *IL10RA* each with 12, *CCND1* with 11, *CSF2RB* and *LIFR* each with 10 and the remaining 10 mRNAs were each associated with 5 or less miRNAs. *CCND1* (FC 2.59), *BCL2L1* (FC 2.25), *MYC* (FC 3.70), along with *CCND2* (FC 1.60) and *SOCS7* (FC 1.51), are all located downstream of STAT activation and were upregulated in carcinoma tissue. As *STAT1* is upregulated in carcinoma tissue (FC 1.55), it could be that these mRNAs are upregulated as a result of increased STAT activity. *PTPN11* was also upregulated in carcinoma tissue, as were the majority of miRNAs associated with this gene. *PTPN11* (tyrosine-protein phosphatase non-receptor type 11) encodes for the SHP2 protein, which is thought to both inhibit JAK-STAT signaling, through inhibition of STAT1, STAT5A and STAT3, as well as enhance it, by preventing SOCS1/JAK2 associations and subsequent STAT5 activation by JAK2 [24]. STAT3 has been proposed to regulate miRNA expression through positive and negative feedback loops and in doing so impact many processes important to development and pathogenesis, including various cancers [11]. Specifically, miR-9, miR-17, miR-19a/b, miR-20a/b, miR-21, miR-155 and miR-181 have been identified as miRNAs involved in STAT3-dependent circuits within various cancers, although not specifically colorectal cancer [11]. We saw decreased levels of *STAT3* (FC 0.91), increased levels of *STAT1* (FC 1.55), and slightly, although statistically insignificant, increased levels of *STAT5B* (FC 1.03), while expression of *STAT5A* remained relatively unchanged (FC 1.00, rounded from 0.9985). *JAK2* expression was downregulated in carcinoma tissue (FC 0.82) and *SOCS1* expression was slightly upregulated in carcinoma tissue (FC 1.05), although this finding was not statistically significant. The only *STATs* that were tested for associations with differential miRNA expression were *STAT1* and *STAT4*, and no significant associations were detected for either mRNA. However, the miRNAs associated with *CCND1*/*2*, *BCL2L1*, *MYC* and *SOCS7* were largely upregulated when the mRNA was upregulated, even when a seed match was identified. *CCND1* was associated with 11 miRNAs, nine of which had seed matches, all of which had positive beta coefficients; *BCL2L1* was associated with one miRNA with a positive beta coefficient and no seed match and *MYC* was associated with 12 miRNAs, 11 of which had positive beta coefficients and none of which had seed matches. These findings suggest that indirect effects, or possibly miRNA transcription activation, are responsible for the differences in expression of these miRNAs.

**Figure 3:**
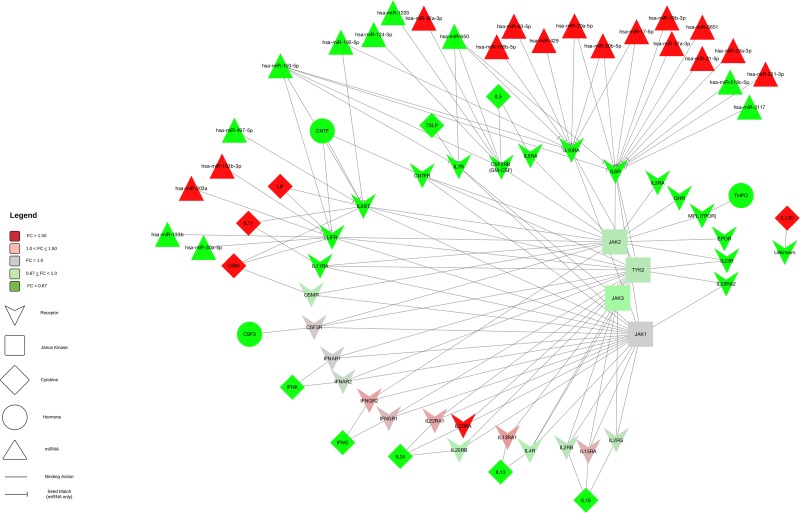
Receptor, cytokine, and hormone interactions and associated miRNAs in the JAK-STAT signaling pathway Figure 3 displays the mRNAs from the JAK-STAT pathway that encode for receptor, cytokine or hormone molecules, as there were too many genes in these categories to include in the overall pathway figure. Note that mRNAs that had a 0.67< FC < 1.50 were included in this figure, to show where they fit in the pathway, even though they were not tested for miRNA associations. FC of the miRNA or mRNA is represented in the color of the molecule: green molecules have less expression in carcinoma tissue compared to normal mucosa and red molecules have higher; paler molecules have a less extreme FC. Also included in this figure are the miRNAs associated with these genes; only miRNAs with a seed region match were included, and these interactions were displayed with a stop (--|).

**Figure 4:**
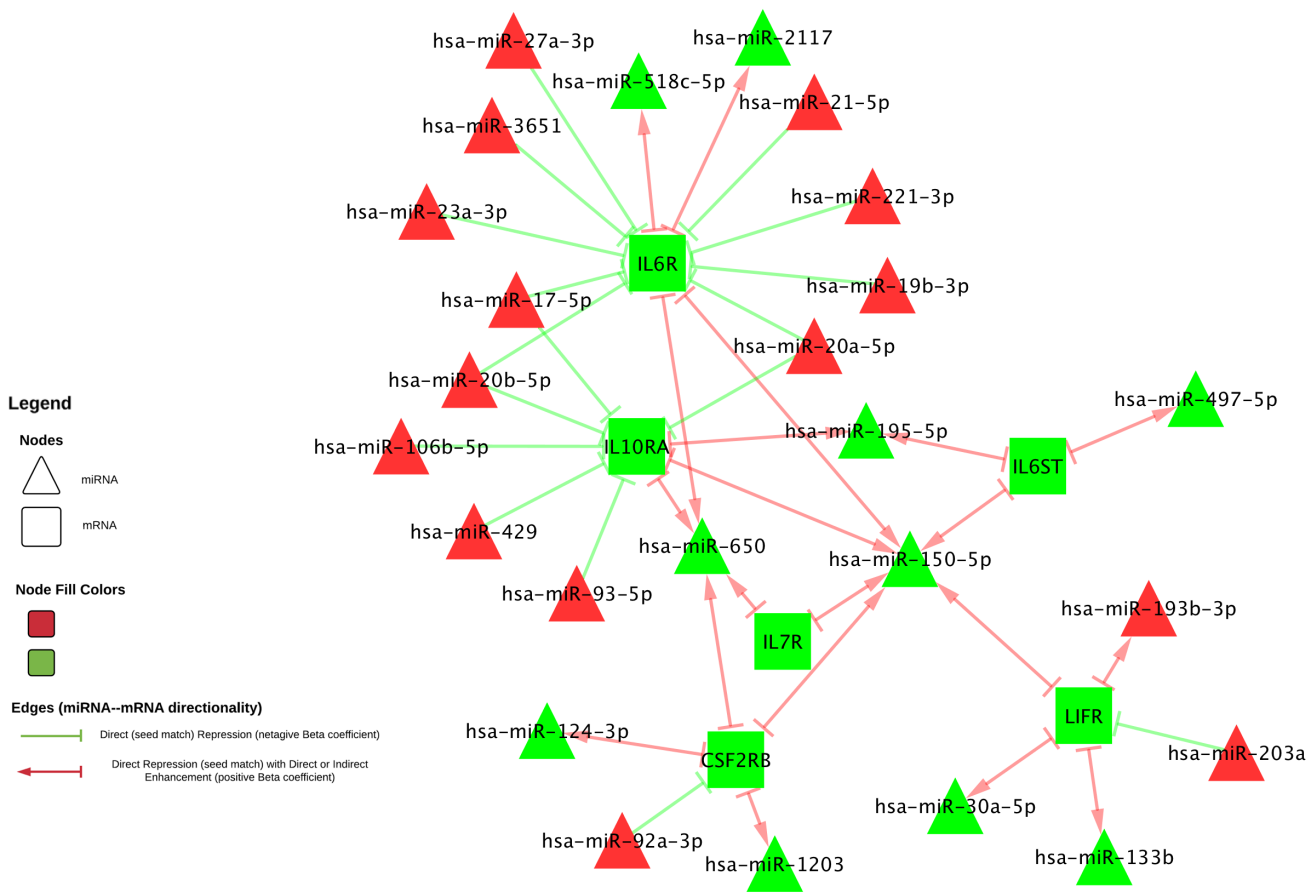
Interactions involving receptor-encoding mRNAs and miRNAs with an identified seed match Figure 4 displays only miRNA-mRNA interactions for receptor-encoding mRNAs with an identified seed match. Interactions with a negative beta coefficient are shown with a green stop (--|) going from the miRNA to the mRNA, and interactions with a positive beta coefficient are shown in red, with an arrow leading from the mRNA to the miRNA and a stop leading from the miRNA to the mRNA. FC of the miRNA or mRNA is represented in the color of the molecule: green molecules have less expression in carcinoma tissue compared to normal mucosa and red have higher.

Genes encoding for receptors accounted for the largest group of dysregulated mRNAs in overall CRC. Similarly, seven of the 17 mRNAs differentially expressed and associated with differential miRNA expression encoded for receptor molecules that bind to JAK at the beginning of the JAK-STAT pathway and, as stated previously, genes that encoded for receptors made up the majority of those that had both a negative beta coefficient and a seed region match, comprising 17 of the total 20 such interactions (see Figure 4). Additionally, these mRNAs were all downregulated in carcinoma tissue compared to normal mucosa, while the miRNAs associated with them were upregulated, supporting the hypothesis that these miRNAs serve to tamp down JAK-STAT activity. Expression of many of the other receptor-encoding genes was also downregulated in carcinoma tissue (see Figure 3). Receptor transcript degradation could inhibit JAK-STAT pathway, as binding to the resulting proteins by ligands initiates the signaling cascade. This supposition is supported by the downregulation of *JAK2* (FC 0.82), *JAK3* (FC 0.71) and *TYK2* (FC 0.84) and the relatively stable expression of *JAK1* (FC 1.01). As the majority of genes downstream of JAK activation in the JAK-STAT signaling pathway were upregulated, however, it is possible that other mechanisms are able to compensate for decreased receptor expression, or receptor activity was only diminished, as a means to maintain homeostasis. JAK-STAT activation stimulates cell proliferation, as well as other potentially metastatic processes, and unchecked stimulation of this pathway may lead to uncontrolled cell growth; JAK-STAT signaling is therefore intended to be transient. As previously stated, miRNAs have been implicated as one of the feedback mechanisms that maintain JAK-STAT homeostasis. Decreased expression of *LIFR* (FC 0.22) was associated with increased differential expression of hsa-miR-203a (FC 3.38), and a seed match was identified between this pair. Similar associations include *CSF2RB* (FC 0.38) and hsa-miR-92a-3p (FC 2.95), *IL10RA* (FC 0.40) and hsa-miR-106b-5p (FC 3.06), hsa-miR-17-5p (FC 3.73), hsa-miR-20a-5p (FC 4.02), hsa-miR-20b-5p (FC 5.35), hsa-miR-429 (FC 1.61), hsa-miR-93-5p (FC 2.74), and *IL6R* (FC 0.26) and hsa-miR-17-5p, hsa-miR-19b-3p (FC 2.86), hsa-miR-20a-5p, hsa-miR-21-5p (FC 2.77), hsa-miR-221-3p (FC 3.28), hsa-miR-23a-3p (FC 2.00), hsa-miR-27a-3p (FC 2.42) and hsa-miR-3651 (FC 2.26), all of which were associated with negative beta coefficients and had identified seed matches. Expression of other receptor molecules (*IL13RA1*, *IL15RA*, *IL22RA1* and *IFNGR1*/*2*) was increased in carcinoma tissue, however due to our FC restriction we did not test these mRNAs for associations with miRNA differential expression. Steady expression, or slight increase of expression, of these receptor genes, as well as expression of genes such as *PTPN11*, may allow the JAK-STAT cascade to continue, while the potentially miRNA-mediated decrease in expression of other receptor molecules serves to rein in uncontrolled growth.

One potential limitation of our study is that we chose to limit our analysis of miRNAs to genes with a FC of >1.50 or <0.67, and as such we did not evaluate JAK-STAT signaling genes whose FC fell outside this range with miRNA expression. Such genes included the *JAK*s (*JAK1* FC 1.01, *JAK2* FC 0.82, *JAK3* 0.71 and *TYK2* 0.84), many of the *STAT*s (*STAT2* FC 0.99, *STAT3* FC 0.91, *STAT5A* FC 1.00, *STAT5B* FC 1.03 and *STAT6* FC 0.97) and many of the *SOC*s (*SOCS1* FC 1.05, *SOCS3* FC 0.83, *SOCS4* FC 1.17, *SOCS5* FC 1.15 and *SOCS6* FC 0.68). This was done to focus our analyses to genes most likely to have a greater biological impact and reduce statistical noise, however expression of genes with smaller FC may also be influenced by miRNAs. As miRNAs have been thought to act as ‘fine-tuners’ of expression and maintain homeostasis, it could be that small changes in expression, while having limited effect on the signaling pathway, do reflect meaningful regulatory interactions [25]. Additionally, we did not see associations involving miRNAs cited in the literature as being enhanced in the JAK-STAT signaling pathway, namely miR-9, miR-21, miR-29a, miR-29b-1 and miR-155. It is possible that these miRNAs are not differentially expressed in CRC, however it may be that these miRNAs would have been associated with mRNAs that were not included in this analysis due to our FC restriction, or that our adjustment for multiple comparisons could have decreased the level of significance from that observed in a targeted miRNA study. We did detect multiple associations with miR-20a-5p and miR-20b-5p, which were identified in the literature as a miRNA involved in STAT3 circuits in breast cancer and gliomas respectively [11].

MiRNAs can regulate many mRNAs and most mRNAs are, in turn, regulated by more than one miRNA. Along with the canonical role of mRNA repression, miRNAs have been implicated more recently in mRNA upregulation, making these interactions more complex and difficult to decipher. In this investigation, we are able to take a discovery approach, by using a large miRNA platform and RNA-Seq; as such, we uncovered many miRNA-mRNA associations in CRC tissue involving genes within the JAK-STAT signaling pathway. This supports the hypothesis that miRNAs are involved in regulating genes at many points in the JAK-STAT signaling cascade in colorectal cancer, and may be responsible for impeding the JAK-STAT pathway by decreasing receptor production. By combining these data along with seed sequence matches, we were able to render a clearer picture of how these molecules interact. However, as many of our associations, including those with identified seed matches, involved a positive beta coefficient, additional investigation involving laboratory experiments are required to determine the exact mechanisms of regulation and whether the effects we see here are the result of direct or indirect enhancement.

## CONCLUSIONS

Many genes within the JAK-STAT signaling pathway are dysregulated in colorectal carcinoma tissue compared to normal colorectal mucosa. Differential expression of 46 miRNAs was associated with the differential expression of 17 of these genes. A negative beta coefficient and seed matches were identified for 20 interactions, indicating these miRNAs target these mRNAs and cause transcript degradation, supporting the hypothesis that miRNAs act to mediate the JAK-STAT signaling cascade. Other miRNAs, which displayed positive interactions with mRNA expression, may themselves influenced by the JAK-STAT pathway.

## MATERIALS AND METHODS

### Study participants

Study participants came from two population-based case-control studies that included all incident colon and rectal cancer between 30 to 79 years of age in Utah or were members of Kaiser Permanente of Northern California (KPNC). Participants were non-Hispanic white, Hispanic, or black for the colon cancer study; the rectal cancer study also included people of Asian race [26, 27]. Case diagnosis was verified by tumor registry data as a first primary adenocarcinoma of the colon or rectum and occurred between October 1991 and September 1994 (colon study) and between May 1997 and May 2001 (rectal study) [16]. The Institutional Review Boards (IRB) at the University of Utah and at KPNC approved the study.

### RNA processing

Formalin-fixed paraffin embedded tissue from the initial biopsy or surgery was used to extract RNA. RNA was then isolated and purified from carcinoma tissue and adjacent normal mucosa as previously described [12]. We observed no differences in RNA quality based on age of the tissue.

### mRNA: RNA-Seq sequencing library preparation and data processing

Total RNA from 245 colorectal carcinoma and normal mucosa pairs was chosen for sequencing based on availability of RNA and high quality miRNA data; 217 pairs passed quality control (QC) and are used in these analyses. RNA library construction was done with the Illumina TruSeq Stranded Total RNA Sample Preparation Kit with Ribo-Zero. The samples were then fragmented and primed for cDNA synthesis, adapters were then ligated onto the cDNA, and the resulting samples were then amplified using PCR; the amplified library was then purified using Agencount AMPure XP beads. A more detailed description of the methods can be found in our previous work [28]. Illumina TruSeq v3 single read flow cell and a 50 cycle single-read sequence run was performed on an Illumina HiSeq instrument. Reads were aligned to a sequence database containing the human genome (build GRCh37/hg19, February 2009 from genome.ucsc.edu) and alignment was performed using novoalign v2.08.01. Total gene counts were calculated for each exon and UTR of the genes using a list of gene coordinates obtained from http://genome.ucsc.edu. We disregarded genes that were not expressed in our RNA-Seq data or for which the expression was missing for the majority of samples [28].

### miRNA

The Agilent Human miRNA Microarray V19.0 was used. Data were required to pass stringent QC parameters established by Agilent that included tests for excessive background fluorescence, excessive variation among probe sequence replicates on the array, and measures of the total gene signal on the array to assess low signal. Samples that failed to meet quality standards were re-labeled, hybridized to arrays, and re-scanned. If a sample failed QC assessment a second time, the sample was excluded from the analysis. The repeatability associated with this microarray was extremely high (*r* = 0.98) [16]; comparison of miRNA expression levels obtained from the Agilent microarray to those obtained from qPCR had an agreement of 100% in terms of directionality of findings and the FCs were almost identical [29]. To normalize differences in miRNA expression that could be attributed to the array, amount of RNA, location on array, or factors that could erroneously influence miRNA expression levels, total gene signal was normalized by multiplying each sample by a scaling factor which was the median of the 75th percentiles of all the samples divided by the individual 75th percentile of each sample [30].

### JAK-STAT signaling genes

The Kyoto Encyclopedia of Genes and Genomes (KEGG) (https://www.genome.jp/kegg-bin/show_pathway?hsa04630) pathway map for JAK-STAT-signaling was used to identify genes associated with this pathway. Using this map, we identified 156 genes (Supplemental Table 1), of which we were able to analyze 122 that were expressed sufficiently in colorectal tissue.

### Statistical methods

We utilized a negative binomial mixed effects model in SAS (accounting for carcinoma/normal status as well as for subject effect) to determine genes in the JAK-STAT signaling pathway that had a significant difference in expression between individually paired colorectal carcinoma and normal mucosa and related fold changes (FC). In this test, we offset the overall exposure as the expression of all identified protein-coding genes (*n*= 17461). The Benjamini and Hochberg [31] procedure was used to control the false discovery rate (FDR) using a value of 0.05 or less. An FC greater than one indicates a positive differential expression (i.e. up-regulated in carcinoma), while an FC between zero and one indicates a negative differential expression (i.e. down-regulated in carcinoma). We determined expression level of each gene by dividing the total expression for that gene in an individual by the total expression of all protein-coding genes per million transcripts (RPMPCG or reads per million protein-coding genes). We focused on those genes with an FC of >1.50 or <0.67 for analysis with miRNAs, under the assumption that these levels of FC may have a greater biological significance than smaller FCs. There were 814 miRNAs expressed in greater than 20% of normal colorectal mucosa that were analyzed; differential expression was calculated using subject-level paired data as the expression in the carcinoma tissue minus the expression in the normal mucosa. In these analyses, we fit a least squares linear regression model to the RPMPCG differential expression levels and miRNA differential expression levels. P-values were generated using the bootstrap method by creating a distribution of 10,000 F statistics derived by resampling the residuals from the null hypothesis model of no association between gene expression and miRNA expression using the boot package in R. Linear models were adjusted for age and sex. Multiple comparison adjustments for gene/miRNA associations were made at the gene level using the FDR by Benjamini and Hochberg [31].

### Bioinformatics analysis

We determined seed region pairings between miRNA and mRNA by analyzing the mRNA 3′ UTR FASTA as well as the seed region sequence of the associated miRNA. As described in our previous work [32], we calculated and included seeds of six, seven, and eight nucleotides in length. A seed match would increase the probability that identified genes associated with specific miRNAs are more likely to have a direct association, given a higher propensity for binding and thus mRNA degradation. As miRTarBase [22] uses findings from many different investigations spanning across years and alignments, we used FASTA sequences generated from both GRCh37 and GRCh38 Homo sapiens alignments, using UCSC Table Browser (https://genome.ucsc.edu/cgi-bin/hgTables) [33]. We downloaded FASTA sequences that matched our Ensembl IDs and had a consensus coding sequences (CCDS) available. Analysis was conducted using scripts in R 3.2.3 and in perl 5.018002.

Cytoscape and the plugin KEGGscape were used to visualize the data [34]. We visualized all molecules involved in miRNA-mRNA interactions and mRNAs that were significantly differentially expressed with a FC >1.5 or <0.67 in overall CRC, rather than for microsatellite stable (MSS) or unstable (MSI) tumors only.

## DECLARATIONS

### Ethics approval and consent to participate

All participants signed an informed consent. The IRBs at the University of Utah and KPNC approved this study; the committee numbers for the data used in this study are IRB_00055877 and IRB_00002335.

## Abbreviations

CRC: colorectal cancer
FC: fold change
FDR: false discovery rate
KEGG: Kyoto Encyclopedia of Genes and Genomes
KPNC: Kaiser Permanente of Northern California
miRNA: microRNA
mRNA: messenger RNA
MSI: microsatellite unstable
MSS: microsatellite stable
QC: quality control
RPMPCG: reads per million protein-coding genes
